# Perspectives on implementing a quality improvement collaborative to improve person-centered care for maternal and reproductive health in Kenya

**DOI:** 10.1093/intqhc/mzaa130

**Published:** 2020-10-15

**Authors:** Katie Giessler, Avery Seefeld, Dominic Montagu, Beth Phillips, James Mwangi, Meghan Munson, Cathy Green, James Opot, Ginger Golub

**Affiliations:** Institute for Global Health Sciences, University of California, San Francisco, Mission Hall, Box 1224, 550 16th Street, 3rd Floor, San Francisco, CA, 94158, USA; Institute for Global Health Sciences, University of California, San Francisco, Mission Hall, Box 1224, 550 16th Street, 3rd Floor, San Francisco, CA, 94158, USA; Institute for Global Health Sciences, University of California, San Francisco, Mission Hall, Box 1224, 550 16th Street, 3rd Floor, San Francisco, CA, 94158, USA; Institute for Global Health Sciences, University of California, San Francisco, Mission Hall, Box 1224, 550 16th Street, 3rd Floor, San Francisco, CA, 94158, USA; Jacaranda Health, Nairobi, Kenya, Diani Close, Nairobi, Kenya; Jacaranda Health, Nairobi, Kenya, Diani Close, Nairobi, Kenya; Jacaranda Health, Nairobi, Kenya, Diani Close, Nairobi, Kenya; Innovations for Poverty Action, Sandalwood Lane, Nairobi, Kenya; Innovations for Poverty Action, Sandalwood Lane, Nairobi, Kenya

**Keywords:** person-centered care, maternal health, family planning, quality improvement, Kenya, patient–provider communication/information

## Abstract

**Objective:**

To understand perspectives and experiences related to participation in a quality improvement collaborative (QIC) to improve person-centered care (PCC) for maternal health and family planning (FP) in Kenya.

**Design and setting:**

Semi-structured qualitative interviews were conducted with members of the QIC in four public health facilities in Kenya.

**Participants:**

Clinical and nonclinical public health facility staff who had participated in the QIC were purposively sampled to participate in the semi-structured interviews.

**Intervention:**

A QIC was implemented across four public health facilities in Nairobi and Kiambu Counties in Kenya to improve PCC experiences for women seeking maternity or FP services.

**Main outcome measure:**

Semi-structured interviews with participants of the QIC to understand perspectives and experiences associated with sensitization to and implementation of PCC behaviors in maternity and FP services.

**Results:**

Respondents reported that sensitization to PCC principles resulted in multiple perceived benefits for staff and patients alike, including improved interactions with patients and clients, deeper awareness of patient and client preferences, and improved interpersonal skills and greater job satisfaction. Respondents also highlighted system-level challenges that impeded their ability to consistently provide high-quality PCC to women, namely staff shortages and frequent turnover, high patient volumes and lack of space in their respective health facilities.

**Conclusion:**

Respondents were easily able to articulate perceived benefits derived from participation in this QIC, although they were equally able to identify challenges that hindered their ability to consistently provide high-quality PCC to women seeking maternity or FP services.

## Introduction

Kenya has made improvements in maternal health (MH) in recent years. The maternal mortality ratio (MMR) halved between 2007 and 2017, from 708 to 342 deaths per 100 000, improving faster and more significantly than the averages for the world as a whole and for all of sub-Saharan Africa [1, 2]. This reduction is likely associated with strategies implemented by the Kenyan Ministry of Health to improve the quality of healthcare received in both private and public sectors [3]. Both policymakers and healthcare providers prioritize identification of effective, relevant and cost-efficient interventions to improve maternal and reproductive health outcomes in Kenya. Despite the impressive reduction in MMR over the past decade, clinical and experiential quality for MH care continues to be challenging [4, 5]. Poor quality of healthcare contributes to women delaying or avoiding seeking care [6]. A recent study of health professionals in Ethiopia found that almost 80% of respondents believed that poor experiential quality during childbirth is a deterrent to seeking delivery care in health facilities [7]. This delay in accessing formalized healthcare for labor and delivery puts women and their babies at increased risk of complications and death [8]. Mistreatment of women during labor and delivery is highly prevalent in the Kenyan healthcare system; one recent study highlighted that 20% of women reported some form of disrespect or abuse during childbirth [9]. Conversely, another recent study found that respondents reported positive experiences of maternity care when it had a person-centered focus, including treating women in a respectful and dignified manner, and ensuring that they were given sufficient information about their care [10]. Person-centered care (PCC) places a patient at the center of their care. For reproductive health services and maternal health care, PCC ensures that a women is actively engaged in decision-making, properly informed about her treatment plan and family planning (FP) options, treated in a kind and dignified manner and is both seen and treated as an individual with unique needs and preferences [11, 12].

One approach that shows promise for improving PCC in low and middle-income countries (LMICs) is the Model for Improvement, a framework that facilitates setting a clear aim, monitoring progress with repeated measurement and testing ideas for improvement sourced from best practice and/or frontline staff [13]. New ideas are only implemented when there is evidence of their efficacy from repeated testing in context. Identification of process or behavior changes that improve performance is accelerated through a quality improvement collaborative (QIC) [14]. Within a QIC, health system stakeholders such as providers and administrators come together to improve a shared set of healthcare-related challenges or outcomes. Quality improvement (QI) teams meet periodically to share their ideas, both successful and unsuccessful. This information is utilized by QIC participants to implement process or behavior changes [14]. Evidence for the QIC approach varies; a recent systematic review reported mixed efficacy in LMICs, highlighting that a QIC fortified by other intervention components, such as training, resulted in better effectiveness of the QIC intervention approach [15]. However, other studies examining efficacy of QICs indicate improvement of maternal, child and reproductive health outcomes specifically in LMICs. In Ghana, implementation of a QIC resulted in improved maternal and child health outcomes, including increases in early adoption of antenatal care and reduction in under-five mortality [16].

Our study focuses on the experiences of both clinical and nonclinical staff who took part in a QIC focused on improving PCC for MH and FP in public facilities in Kenya. We hypothesized that participation in the QIC and/or sensitization to PCC principles would have benefits for both providers and their patients: women seeking maternity and/or FP services, hereinafter referred to as ‘women.’ At the same time, we hypothesized that systemic barriers could prevent comprehensive and lasting implementation of PCC behaviors within study facilities. Results on the ability to improve PCC within the framework of a QIC are reported elsewhere [17].

## Methods

### Quality improvement collaborative

Four public health facilities in Nairobi and Kiambu Counties were selected to participate in an intervention focused on improving PCC for MH and FP delivered through a QIC. Intervention sites were selected based on reported patient volumes of at least 100 deliveries per month and a reported mix of FP method provision and endorsement by senior leadership within local government. Sites were excluded if they had participated in PCC initiatives previously. Sites ranged in size and capacity from smaller health centers to county-level referral hospitals. Performance indicators at baseline among all intervention facilities were used to prioritize PCC intervention topics. These included consistently using women’s names when speaking to them, self-introduction by the provider and explaining tests, medicines and procedures. QI teams comprised mostly of FP and maternity trained clinicians, in addition to administrative staff such as data clerks and health records officers, were formed in each intervention facility. QI teams were supported throughout the QIC by an experienced facilitator from a local non-governmental organization that had been trained extensively in QI methodology by an external QI expert that designed the QIC intervention. The QI facilitator met weekly with QI team members in their respective facilities, for an average of 54 meetings per site. Facility data clerks and health records officers at each site aimed to conduct 10 exit interviews a week with women who had delivered in the facility, as well as 10 exit interviews weekly with women who had obtained FP services. Data clerks and health records officers were selected to conduct exit interviews with women to avoid any response bias that could occur if clinicians who provided care to participating women were to conduct the interviews. Data from exit interviews were used internally by the QI team to understand progress toward an improvement aim identified, for example, that 95% of all women who received delivery services were called by their name. QI teams gathered within the QIC every 3 months across a 9-month period to share progress, present and review data from patient exit interviews for mutual learning and explore topics for improvement in the subsequent quarter. The QI facilitator provided light-touch support via ad hoc meetings with QIC members for an additional 5 months following completion of the QIC in order to sustain improvements gained during the intervention.

### Participant sampling

A descriptive qualitative exploration of QIC participant attitudes and experiences using semi-structured interviews was conducted with QI team members to understand benefits and challenges of participation in the QIC itself, and the experiences of these implementers in incorporating PCC behaviors into their care. Among the 4 facilities, 38 QI team members participated in the intervention; 32 were purposively sampled for in-depth interviews, prioritizing active meeting attendance.

### Data collection procedures

Semi-structured interview guides were developed jointly by the lead researchers, the implementing partner and the evaluation team. Topics included respondents’ definitions and current status of PCC at their facilities, perceptions of women’s expectations of quality care, challenges to providing quality clinical and PCC, experiences implementing the QIC activities and opinions on how quality care can be assured and sustained.

Following the conclusion of the QIC, two researchers experienced in qualitative research underwent a 1-week training on qualitative methods and familiarization with the interview guide through role plays. Researchers then held 1-week of piloting to practice interviewing and refine guides to improve question flow and wording.

Prior to each interview, these researchers sought written consent in the respondent’s preferred language of English or Kiswahili. Interviews were conducted in a private setting within the facility. After each interview, respondents received ∼$1.50 in mobile airtime as a token of appreciation. Data collection took place from November 2018 to February 2019. The audio-recorded interviews were transcribed and simultaneously translated to English, if needed, by an external firm. Transcripts were back checked by the evaluation team to assure quality.

### Data analysis

Using a thematic content analysis approach [18], the researchers developed a codebook based on key themes of the process evaluation. Next, four coders individually coded the same interview, identified categories within themes and compared their analyses. Any intercoder discrepancies were discussed and revised based on team consensus. Two additional interviews were subsequently coded and compared by three of the four initial coders to allow for further codebook refinement. The remaining transcripts were then divided among the four researchers to single code. The team met regularly to discuss questions or emergent themes and resolve concerns based on consensus. Data were analyzed using Atlas.ti, version 8.

### Ethical considerations

The protocol and interview guide were approved by Kenya Medical Research Institute’s Scientific and Ethics Review Unit (Protocol Non-SSC 526), as well as the University of California, San Francisco’s Institutional Review Board (Protocol number 15-18008).

## Results

All 32 QI team members consented to participate in the semi-structured interviews, which lasted 50 minutes on average. Most respondents were nurses/midwives (62.5%). The majority were female (87.5%), with a median age of 40 years. Table 1 specifies respondent demographics.

**Table 1:** Characteristics of active and semi-active members of the quality improvement collaborative

Respondent characteristic	*n* (%)
Gender	
Male	4 (12.5)
Female	28 (87.5)
Age (years): median (IQR)	40 (18.5)
Position	
Clinical officer	2 (6.25)
Nurse/midwife	20 (62.5)
Auxiliary nurse midwife	2 (6.25)
Health records information officer	4 (12.5)
Social worker	2 (6.25)
Data clerk intern	1 (3.13)
Cook	1 (3.13)
Training received	
Certificate	4 (12.5)
Diploma	21 (65.63)
Degree	7 (21.88)
Years in position: median (IQR)	11 (19)
Years at facility: median (IQR)	4.5 (7)

IQR, interquartile range.

### Perceived patient benefits

Some respondents reported that implementing PCC behaviors improved rapport with women, setting the stage for more collaborative interactions and trust between women and providers. These respondents highlighted that calling a patient by her name provided an opportunity for women to be acknowledged as individuals rather than generic ‘patients,’ leading to improved communication between women and providers.

‘I think like we said earlier it is all about giving our clients the best care, individualized care. What one client may require is not what another will require…They are able to ask you questions that they may not have asked earlier on because they feel that you are listening’—Female Nurse/Midwife, 36 years old

By creating this type of rapport, respondents perceived that women acted more in partnership with their providers by being more willing to share information about their health issues and concerns.

‘[Women] were able to open up even in things which they didn’t come for… Because maybe there is something she couldn’t tell me before when I call her “mother.” But now she has felt that I know her, she could open and say everything that she has.’—Female Auxiliary Nurse Midwife, 39 years old

Some respondents reported observing that PCC behaviors, such as explaining the purpose of procedures and providing women with information about their care, helped women to be better prepared for labor and delivery.

‘…also explaining procedures, especially in maternity…[w]hat to expect explaining procedures brings a lot of comfort and confidence to the clients…’—Female Nurse/Midwife, 55 years old

Respondents consistently shared that their training in PCC helped them to provide more individualized care, outlining that this approach facilitated greater trust between women and providers and observed improvements in women-provider interactions.

‘…[T]he importance of calling that client by name, the trust that she has in me when I call her by name and when I call her “mother”…[T]hese are totally two different people.’—Female Nurse/Midwife, 52 years old

### Perceived provider benefits

Multiple respondents also indicated that sensitization to PCC principles led to personal benefits. These included improved interpersonal skills, greater self-efficacy and improved confidence in their professional abilities. One respondent highlighted that he related better to both clients and his colleagues as he grew more familiar with PCC concepts:

‘…I learnt how to relate with others, it has improved my interpersonal relationships skills and it makes me aware of my surrounding and the people I serve and it has also helped me have confidence in mentoring others because there is no disappointment in offering quality.’—Male Clinical Officer, 36 years old

This increased familiarity with PCC concepts and employing such behaviors was perceived as a key learning from respondents’ participation in the QIC. One respondent recalled this when asked about her experience being a QI team member:

‘I have learnt something new. […Over time,] we have come to know that all the areas that we have worked on, they were areas that patients felt they were important. We didn’t think they were in our setup. But now along the way we have noted there is a change. We have noted they really mattered even to our patients.’—Female Nurse/Midwife, 36 years old

Another respondent highlighted that participating in the QI intervention led to a deeper understanding and awareness of patient preferences, resulting in greater connection between providers and women:

‘… I’ve realized with what we have improved with, the calling of the name to the client makes the patient feel so near to you than when you call them by maybe “You, wewe, mama” or something of the sort. They usually don’t like it when you call them as mamas. They prefer being called by their names. And that has brought them closer to us as nurses or the attendants.’—Female Nurse/Midwife, 52 years old

Several respondents also indicated that they felt more satisfaction and pride in their work following participation in the QIC. One respondent shared that her ability to meet women’s needs as a result of integrating PCC behaviors brought notable gratification to her own work experience:

‘They [facility staff] are able to know and to see the impact they make on a patient so that will motivate them to do the same to others … just seeing the impact that has on a patient and working to improve and to make it better… I think I had mentioned that it gives you a genuine fulfillment on what you are doing and also makes you want to do more … after seeing what patients have to say about it.’—Female Nurse/Midwife, 56 years old

Through participation in the QIC, participants gained familiarity with PCC principles and also found that they received benefits beyond those which were expected as noted above. These included increased confidence in mentoring others in their skillsets, improved clarity related to patient preferences and greater job satisfaction.

‘Well, the benefits as a person…you feel job satisfaction when you know you have given your best. And QI has helped me do that…[m]ost of the things I didn’t know make a difference for a patient have now been [routinely] incorporated into the program. So at the end of the day, you feel you have given that patient the very best so you have job satisfaction. You also feel that you have exhausted everything that you could have done for her.’—Female Nurse/Midwife, 36 years old

### System-level challenges

Although many respondents easily identified intervention benefits, they equally emphasized that the Kenyan health system impedes easy and consistent implementation of PCC. Many providers shared that staffing shortages were the main challenge to implementing PCC. They pointed to disproportionate staff-to-patient ratios that meant respondents could not spend adequate time with a woman to provide PCC.

‘You might be in maternity, you are alone, you have around 5 mothers who want to give birth at the same time. At times you even don’t have time to explain to them what is expected of them, so by the time you are finishing with this one, there is another mother here calling you to come.’—Female Nurse/Midwife, 31 years old

Some respondents also noted that staff turnover and rotations hinder consistent PCC behaviors. Respondents explained that frequently changing staff meant they were often sensitizing new staff to PCC practices, making consistent provision of quality PCC difficult.

‘…somebody has been doing it very well; another day, another group came and maybe you are not there and maybe somebody else forgot to induct to whatever is going on.’—Female Nurse/Midwife, 55 years old

A few respondents emphasized that the time required to attend QI meetings prevented them from fully participating in the intervention given their requisite clinical duties.

‘Maybe we need to be in the QI [meeting] and I have a line in family planning. Remember I am improving the quality of this client, do I leave the client and go for QI? No. So first of all I finish with the client so that I can go to the QI.’—Female Auxiliary Nurse Midwife, 39 years old

Some respondents highlighted lack of space as a challenge to providing PCC, particularly relating to involving labor and birth companions. Women often needed to share beds, and companions could not accompany women during labor and delivery.

‘When a mother is in labor, okay in QI this patient is supposed to be escorted either by her husband, mother, guardian or anybody. But like now in our facility when we receive patients we dismiss these others home. […] Our space is not enough so if we would accommodate everyone who would accompany this patient the place would be very full.’—Male Clinical Records Officer, 40 years old

While respondents were eager to share benefits of the QI intervention, they also highlighted various system-level challenges that complicated the effective execution of such work.

## Discussion

As hypothesized, our findings highlight that implementation of PCC resulted in perceived benefits for both women and providers. Specifically, participation in the intervention changed provider attitudes and led to the QIC members perceiving improvements in provider–women interactions. This finding was particularly notable as PCC principles were not universally accepted as relevant or necessary by all members of the QIC at the outset of the intervention. Specifically, the act of introducing oneself to women was of concern to QIC members given that a culture of blame is pervasive in the public healthcare system in Kenya, and Kenyan healthcare providers are often held responsible for adverse outcomes that are out of their control [19]. Therefore, the authors assume that respondents participating in our QIC may have had reservations about introducing themselves for fear of making themselves potential targets. Yet our study respondents indicated that they perceived a positive effect on the woman’s and provider’s experience of receiving and providing care, respectively, when employing better communication skills, such as introducing themselves to women. Long-term sensitization to specific PCC behaviors such as self-introduction, and greater familiarity with the QI process generally led respondents to indicate that they eventually felt more at ease with this practice, changing the perspective of providers from self-defense to self-efficacy related to the way that they interact with women. Interestingly, self-introduction was identified by multiple respondents as a PCC behavior that had an almost immediate positive impact on their interactions with women.

Respondents also reported experiencing benefits from participation that extended beyond the scope of the project, including improved communication, increased job satisfaction and greater self-confidence. They also emphasized systemic challenges that constrained their ability to deliver high-quality care, irrespective of improvements in their PCC attitudes or the improved interpersonal experiences with women [20]. Systemic constraints identified by respondents focused largely on insufficient staffing numbers and high patient volumes within their respective facilities, with a disproportionate burden falling on clinical staff to provide high-quality care without sufficient resources related to time, physical space and appropriate staff-to-patient ratios. Although such constraints were beyond the scope of the QIC intervention to comprehensively ameliorate, efforts were made by QIC members and the external QI facilitator to reduce constraints that were within their control. For example, QI meetings were arranged to occur on days when the facility was less busy and providers had decreased demands on their time. Generally, nonclinical QIC members were less time-constrained overall and able to participate more consistently in weekly QI meetings.

At the outset of the QIC, the authors hypothesized that many of the PCC principles that were the focus of the intervention could circumvent the systemic constraints described above, as the intervention focused on behavioral change. However, respondents easily identified that poor staff-to-patient ratios significantly impeded their ability to provide high-quality PCC, as demonstrated in the following quote from a female nurse/midwife, 46 years old:

‘…according to WHO one is supposed to attend to six patients per day. But now you find that maybe at times you report to work alone. Maybe you are two with a total of ninety patients. What is that ratio? It is very much right to give it (PCC) [but] that it is not possible.’

Quantitative study results from our project have shown that despite providers reporting improved interactions with women and buy-in with the QI work itself, there was no quantifiable behavior change [17]. This discrepancy may be due to acceptance of PCC principles themselves, as some QIC members did not immediately accept these concepts at the onset of the intervention. Anecdotally, many QI team members did not initially see the value or relevance of implementing PCC principles, indicating that their primary responsibility was to ensure positive clinical outcomes, irrespective of the woman’s experience of care. It is also possible that respondent bias informed our results, with providers describing positive attitudinal changes that were exaggerated. This lack of behavior change may alternatively indicate that when under stress with limited staffing, or when facing emergency situations with inadequate space and supplies, providers revert to habitual behaviors rather than practicing newly learned PCC behaviors [21]. Frequent staff turnover, especially among the QIC participants, may have also contributed to a lack of continuity in PCC behaviors and effective outreach to their peers.

There were several limitations to this study. First, our data are subject to both courtesy and social desirability biases [22, 23]. Respondents may have shared views more socially acceptable than their own to avoid offending the researchers, and over-reported favorable perspectives and under-reported negative ones. To mitigate these biases, we engaged external interviewers unconnected to the implementing partner. Interviewers clearly introduced themselves as unaffiliated prior to asking any questions, but such biases may still be present. Additionally, since only four facilities participated in this study, our results do not generalize to other facilities who may partake in a similar intervention. Moreover, because the interview guide questions focused on the QIC itself, control facility respondents could not participate; therefore, we are unable to compare with this group. Additionally, our respondents were providers who participated in the QIC and not the client beneficiaries of the initiative. Therefore, our data represent perceived benefits for women but may not represent actual benefits received. Lastly, there were external challenges during the intervention that led to imperfect implementation of the QI work. For example, there were multiple nationwide healthcare provider strikes during the QIC, which interrupted normal service delivery and halted QI activities periodically. This disruption led to the QIC lasting longer than is typical, which may have negatively impacted staff motivation and overall implementation.

Our study is among the first of its kind. As the QI process encourages contextual adaptation and adoption of best practice utilizing the knowledge and ideas of frontline workers, the QIC recruited staff from within four health facilities. While research exists on the effects of QIC-led improvement interventions on their primary outcomes, little research has been conducted on how they were perceived from the perspective of the providers themselves.

This study demonstrates the benefits that the QIC in Kenya had on providers’ perspectives and attentiveness to women’s experiences and in the process changed for the betterment of their own experience of delivering care. The positive effect on providers is an encouraging finding as it may speak to the sustainability of changes.

However, respondents were emphatic that larger structural constraints—overworked staff at all levels with high turnover rates and overwhelming patient loads—severely impacted their ability to provide what they felt to be high quality care. Improvements in empathy, communication and engagement with women did not supersede these health system challenges.

Our findings provide hope that healthcare providers can develop skills such as empathy, communication and self-efficacy applying QI methods, which can also enhance job satisfaction despite resource constraints. The Kenyan national policies on advancing Universal Health Coverage and expanding access to MH, exemplified by the 2015 National Hospital Insurance Fund Act, both include provisions to address staffing and provider–patient ratios [24, 25]. If those efforts are successful, our findings indicate potential for further improvement in PCC. More research must be done to determine whether recipients of PCC (women and others seeking healthcare) experienced the perceived additional benefits that respondents highlighted, and whether providers own behavioral changes are sustained beyond any initial change intervention.

## Data Availability

All data are available from the first author upon request.
